# Benefits of VISION Max automated cross-matching in comparison with manual cross-matching: A multidimensional analysis

**DOI:** 10.1371/journal.pone.0226477

**Published:** 2019-12-23

**Authors:** Hee-Jung Chung, Mina Hur, Sang Gyeu Choi, Hyun-Kyung Lee, Seungho Lee, Hanah Kim, Hee-Won Moon, Yeo-Min Yun

**Affiliations:** 1 Department of Laboratory Medicine, Konkuk University Medical Center and Konkuk University School of Medicine, Seoul, South Korea; 2 Department of Occupational and Environmental Medicine, Ajou University Medicine, Suwon, South Korea; Inselspital Universitatsspital Bern, SWITZERLAND

## Abstract

**Background:**

VISION Max (Ortho-Clinical Diagnostics, Raritan, NJ, USA) is a newly introduced automated blood bank system. Cross-matching (XM) is an important test confirming safety by simulating reaction between packed Red Blood Cells (RBCs) and patient blood *in vitro* before transfusion. We assessed the benefits of VISION Max automated XM (A-XM) in comparison with those of manual XM (M-XM) by using multidimensional analysis (cost-effectiveness and quality improvement).

**Materials and methods:**

In a total of 327 tests (130 patients), results from A-XM and M-XM were compared. We assessed the concordance rate, risk priority number (RPN), turnaround time, hands-on time, and the costs of both methods. We further simulated their annual effects based on 37,937 XM tests in 2018.

**Results:**

The concordance rate between A-XM and M-XM was 97.9% (320/327, kappa = 0.83), and the seven discordant results were incompatible for transfusion in A-XM, while compatible for transfusion in M-XM. None of the results was incompatible for transfusion in A-XM, while compatible for transfusion in M-XM, meaning A-XM detect agglutination more sensitively and consequently provides a more safe result than M-XM. A-XM was estimated to have a 6.3-fold lower risk (229 vs. 1,435 RPN), shorter turnaround time (19.1 vs. 23.3 min, *P* < 0.0001), shorter hands-on time (1.1 vs. 5.3 min, *P* < 0.0001), and lower costs per single test than M-XM (1.44 vs. 2.70 USD). A-XM permitted annual savings of 46 million RPN, 15.1 months of daytime workers’ labor, and 47,042 USD compared with M-XM.

**Conclusion:**

This is the first attempt to implement A-XM using VISION Max. VISION Max A-XM appears to be a safe, practical, and reliable alternative for pre-transfusion workflow with the potential to improve quality and cost-effectiveness in the blood bank.

## Introduction

The importance of pre-transfusion tests, including cross-matching (XM), is the same as that of pre-transplantation laboratory tests; the importance of XM test, however, is easily underestimated because blood transfusions are routinely performed daily at the blood bank [1]. XM is an important pre-transfusion test confirming the compatibility of blood component for transfusion by observing the antigen-antibody reaction between blood component and patient blood in vitro [2,3]. If the patient is positive in unexpected antibody screening (ABS), the laboratory should identify the unexpected antibody so that they can issue compatible blood component for the patient when there is a transfusion order [3]. Electronic XM (also called computer XM) and automated XM are applied in some countries [4,5]. However, the policy of blood transfusion and blood supply varies greatly from country to country [4,5].

The result of the error in pre-transfusion tests can be critical or fatal [6,7]. Spillage or a small amount of the sample during XM tests may result in a re-examination and a delayed examination, and an error such as mislabeling of the patient sample may lead to an inadequate blood transfusion, even leading to patient death [7–9].

Recently, "patient safety" has been increasingly emphasized in healthcare, and efforts to prevent adverse events by reducing risk have been actively pursued [10–12]. From 2002, the Joint Commission on Accreditation of Healthcare Organizations (JCAHO) began to mandate proactive risk assessment using failure mode and effects analysis (FMEA) to reduce the risk before an adverse event [13]. FMEA has been used in high-risk industries, such as in the astrospace sector and has been proved to be promising in reducing the risk of errors in the medical field [14,15]. In laboratory medicine, including transfusion medicine, the FMEA model is a useful tool in proactively analyzing and reducing the risks [16–21]. While reducing risk and reporting accurate results, it is also necessary to report the test results promptly to maintain the high quality of laboratory tests [22]. The FMEA model is already adopted in blood transfusion area to reduce risk and increase patient safety [16–18], and using the FMEA we previously reported benefits of automation in blood bank [17].

VISION Max (Ortho-Clinical Diagnostics, Raritan, NJ, USA) is a newly introduced automated blood bank system that is based on an agglutination method using a column containing a glass microbead matrix [23]. VISION Max automates the full range of immunohematologic testings, including ABO/Rh typing, XM, direct antiglobulin testing (DAT), ABS/antibody identification, and antigen testing [23,24]. Its middleware system is highly flexible and can be customized to each hospital's laboratory information system (LIS) [23,24].

Laboratory automation is an irreversible big trend [25–27]. Although automation of pre-transfusion testing processes can dramatically reduce error potentials and improve the safety of blood transfusion [28], clinical studies on automated XM (A-XM) is very limited [29,30]. In this study, we adopted VISION Max A-XM with customized middleware system and explored the benefits of using VISION Max A-XM in comparison with manual XM (M-XM) by multidimensional analysis. To assess performance, we observed the concordance rate; to assess quality, we observed the risk, turnaround time (TAT), and hands-on time; and to assess the effectiveness, we estimated costs. To the best of our knowledge, this is the first study on A-XM evaluation using VISION Max.

## Materials and methods

### Study design

This study was conducted in Konkuk University Medical Center (KUMC), a 700-bed tertiary-care teaching hospital, according to the Declaration of Helsinki. The protocol was approved by the Institutional Review Board of KUMC (KUH12000111), with an exemption of obtaining informed consent from the participants [31]. This reporting followed the STARD guidelines [32]. A-XM and M-XM were performed simultaneously with split-sample, and both results cannot be referred to the medical technician. After user education, training, and 10 weeks of familiarization period to provide sufficient experience to the medical technician, we enrolled all the consecutive clinical samples (regardless of age, gender, department, and patient setting) that were sent to the blood bank for routine XM testing between 2 pm and 4 pm from March 2 to March 31, 2018. Enrolled sample group in this experiment was exactly the same setting with the whole sample group undergoing XM test in the clinical setting. Because the subject of this paper is a sample, not a patient, sample eligibility followed the specimen rejection criteria of laboratory standard operation manual for XM testing of KUMC (S1 File). Laboratory step by step protocols are shared in protocol.io (dx.doi.org/10.17504/protocols.io.652hg8e) according to the instruction for the author. Specimen rejection criteria were: patient sample older than 24 hours from specimen collection, patient sample without appropriate labeling (patient’s identification number in hospital/name/age/gender and name of phlebotomist), hemolyzed sample by visual inspection, and not enough specimen less than 1.5 mL as serum/plasma (S1 File). Commercially available BioVue Screen Panels (Ortho-clinical Diagnostics, Raritan, New Jersey, USA) was used for ABS. Antibody identification was done in ABS positive samples, using 0.8% ORTHO RESOLVE Panel (Ortho-clinical Diagnostics) which is composed of 11 cells.

VISION Max provides flexible middleware, which bi-directionally communicates with the LIS and the analyzer [23,24]. The middleware is customizable to the situation of each hospital and blood bank, using tailored reflex test, automated verification, and responsive automation. VISION Max automatically accumulates all the compatible/incompatible XM results and provides statistics. For discordant results, results of ABO/Rh typing, ABS/antibody identification, DAT, and history of blood transfusions within three months were reviewed. We further simulated their annual effects based on the test numbers in 2018.

### Cross-matching and concordance rate

A total of 327 samples (from 130 patients) were used to compare the results between A-XM and M-XM. The necessary sample size was 73 which was calculated based upon the accumulated incompatible rate 5.0% of our laboratory (95% confidence level, 0.05% of margin of error) by Cochran’s sample size recommendation [33]. Venous whole blood (6 mL) was drawn into BD Vacutainer serum tube (Becton Dickinson and Company, Franklin Lakes, NJ, USA), and serum was separated by centrifuging at 1000 g for 10 mins in room temperature.

A-XM was performed in VISION Max, using a Poly Cassette (Ortho-Clinical Diagnostics) including anti-IgG and anti-C3d. Briefly, 500 uL of donor red blood cells (RBCs) is added to the test tube, and the test tube and the plasma of the recipient are placed into the rack and then placed in the VISION Max. The barcode of the blood component is scanned. Thereafter, the robotic pipetting arm inside VISION Max automatically transfers 50 uL of BLISS, 40 uL of plasma, and 10 uL of 3% RBCs to Poly Cassette. The Poly Cassette is incubated at 37°C for 10 minutes and centrifuged for 5 minutes. Results of agglutination are determined as 0, ±, +1, +2, +3, and +4. The camera inside VISION Max reads the column and records agglutination grade with a digital image. Negative results of A-XM are assigned as compatible, and all the other results are assigned as incompatible. In a batch, seven XM tests can be performed simultaneously.

M-XM was sequentially tested through the 1st saline phase, 2nd albumin phase, and 3rd anti-human globulin phase. The M-XM process was performed according to the laboratory standard operation manual of KUMC (S1 File) based on Technical Manual of American Association of Blood Banks [2]. All XM tests were performed by the same experienced medical technician and were determined as compatible or incompatible. The concordance rate was calculated by the proportion of concordant pairs over the total number of paired tests. [34]. The concordance rate of A-XM and M-XM results was assessed overall and according to the presence of unexpected Ab, that is ABS positivity. Because unexpected Ab in patient blood more often produces incompatible results in XM with donor blood component. Therefore the authors expected that the concordance rate of A-XM and M-XM results will be higher [3].

### Risk assessment by FMEA

According to the international standard for FMEA, the risk priority number (RPN) was calculated to quantify risks as following formula: RPN = severity (S) × occurrence (O) × detection (D) [35]. All three medical technicians working at the blood bank, one attending medical doctor, and one quality manager participated in the FMEA and RPN scoring [35]. During A-XM and M-XM processes, each step of workflow was separated, described, and reviewed. In each step, possible failure modes and subsequent potential effects were described based on the laboratory logbook, troubleshooting records, and interviews based on our previous FMEA assessment study in transfusion area [17] and International Standard for FMEA [35]. Based on this, rating score and operational definitions of severity of failure, frequency of occurrence, and detection of failure were determined (Table 1) [17]. As an example of RPN scoring, mis-recording of M-XM result happens with a frequency of less than once a year in our laboratory. In this event, severity score is 10 and the occurrence score is 1 and because all the M-XM results are cross-checked by a different medical technician, the detection score is 7, according to Table 1. So, the calculated RPN score of this event is severity (10) × occurrence (1) × detection (7) = 70.

**Table 1:** Rating score and operational definitions of severity of failure, frequency of occurrence, and detection of failure considered to calculate RPN in FMEA in blood bank tests [17].

Score	Severity of failure	Score	Occurrence	Score	Detection
**1**	Unnoticed, no relevant effect	**1**	< once a year	**1**	Clearly visible, 100% instrumental inspection with preventive maintenance
**2**	Failure not unnoticed, little effect, very minor nuisance	**2**	< twice a year	**2**	100% instrumental inspection with preventive maintenance
**3**	Creates extra effort, minor nuisance	**3**	< once a month	**3**	100% instrumental inspection
**4**	Creates some rework	**4**	< once a week	**4**	Partial instrumental inspection
**5**	Creates moderate rework	**5**	< once a day	**5**	Periodic inspection by laboratory personnel
**6**	Creates considerable rework	**6**	≥ once a day or more often	**6**	100% manual inspection and has visual cues
**7**	Causes considerable and possible instrument downtime rework			**7**	100% manual inspection
**8**	Could be reportable error and possible instrument downtime			**8**	Random manual inspection or a low chance of detection
**9**	Could harm a patient but may not cause death and/or possible instrument failure			**9**	Random manual inspection or a low chance of detection
**10**	Could injure a patient or cause death and/or possible instrument failure			**10**	No inspection or no chance of detection

Abbreviations: RPN, risk priority number; FMEA, failure modes and effects analysis.

In this study, 600 RPN was the highest score, which indicated that patients could be injured or even died every day.

### TAT and hands-on Time

The TAT was defined as the time from the point at which XM testing was started to the time point at which the verified result was reported to the hospital LIS. To measure TAT, XM testing process was performed seven times and was recorded as a video; the video records were analyzed in seconds for TAT in each process step. Hands-on time was defined as the time taken by medical technicians for XM testing; it was calculated by subtracting the sample processing time in the Vision Max from the TAT.

### Cost-effectiveness

The total costs included direct and indirect costs. Direct costs included costs for reagents and consumables per single test in A-XM and M-XM. Instrument cost was included in the direct cost as lease contract. Indirect costs included depreciation costs and labor costs. Depreciation costs were taken into account only when the instruments were directly related to the tests and purchased within the last five years [36]; as any of the instruments in the present study were not purchased within the last five years, depreciation costs were not included. Labor costs were calculated on the basis of the average operator salaries and hands-on time. Labor costs may vary across institutions; therefore, it was calculated by referring to the published multi-center research paper in Korea, considering 3.0% of the annual domestic inflation rate [36].

### Statistical analyses

The Cohen's kappa was used to assess agreement between the two categories [37]. Homogeneity of variance was tested by performing F-test and Wilcoxon rank-sum test was used to evaluate the differences of TAT and hands-on time between A-XM and M-XM processes [34]. The data with non-parametric distribution were presented as median and interquartile range [38]. RPNs between A-XM and M-XM processes were relatively compared because RPN has no standard. The level of significance for all statistical analyses was set to *P* < 0.05. Statistical analyses were performed using Microsoft Excel 2016 (Microsoft Corp., Redmond, WA, USA) and R-3.3.2 under CentOS Linux 7.

## Results

### Concordance rate between A-XM and M-XM

The concordance rate between A-XM and M-XM was 97.9% (320/327, kappa = 0.85), showing near perfect agreement (Table 2). The seven discordant results were all from different patients. All the seven cases showed incompatible for transfusion in A-XM testing, while showed compatible for transfusion in M-XM testing. None of the results was incompatible for transfusion in A-XM while compatible for transfusion in M-XM, meaning A-XM detect agglutination more sensitively and consequently provides at least equally safe results with M-XM. Grades of incompatible results with A-XM are shown in **S1 Table**. Among the seven discordant results, three cases (43%) had a transfusion history within 90 days. All the seven discordant cases were negative for auto-antibody. DAT was requested only in two cases, and both of them showed positive results. The ABS positivity was 29% (2/7), having anti-Le^a^ antibody and anti-P_1_ antibody, respectively. Anti-Le^a^ antibody and anti-P_1_ antibody are IgM antibodies rarely the destruction of red blood cells even though presence of those specific antigens. Consequently rarely causing hemolytic transfusion reaction or hemolytic disease of the newborn. We did not evaluate the presence of those specific antigens, respectively. The discordant rate in the ABS-positive group was three times higher than that in the ABS-negative group (4.5% [2/44] vs. 1.8% [5/283]), as expected. In ABS positive group, 14 cases showed incompatible results in both A-XM and M-XM. Their antibody specifications were as follows: anti-E (4 cases), anti-M (3 cases), anti-Le^a^ (2 cases), anti-Le^b^ (2 cases), anti-P_1_ (1 case), and unidentifiable antibody (2 cases).

**Table 2:** Comparison between A-XM and M-XM. Discordant results have shown in bold.

Results			A-XM, n (%)				Total, n (%)		Concordance, n (%)	kappa value (95% CI)
			Compatible		Incompatible					
Overall (n = 327)										
	M-XM	Compatible	302	(92.4)	**7**	**(2.1)**	309	(94.5)	320 (97.9)	0.85 (0.73–0.98)
		Incompatible	**0**	**(0.0)**	18	(5.5)	18	(5.5)		
		Total	302	(92.4)	25	(7.6)	327	(100.0)		
ABS positive (n = 44)										
	M-XM	Compatible	28	(63.6)	**2**	**(4.5)**	30	(68.2)	42 (95.5)	0.90 (0.76–1.00)
		Incompatible	**0**	**(0.0)**	14	(31.8)	14	(31.8)		
		Total	28	(63.6)	16	(36.4)	44	(100.0)		
ABS negative (n = 283)										
	M-XM	Compatible	274	(96.8)	**5**	**(1.8)**	279	(98.6)	278 (98.2)	0.61 (0.29–0.92)
		Incompatible	**0**	**(0.0)**	4	(1.4)	4	(1.4)		
		Total	274	(96.8)	9	(3.2)	283	(100.0)		

Abbreviations: A-XM, automated cross-matching; M-XM, manuals-matching; ABS, antibody screening; CI, confidence interval.

### Comparison of quality and cost between A-XM and M-XM

The A-XM and M-XM processes were determined to have 8 and 18 steps, respectively (Table 3). A-XM was estimated to have a 6.3-fold lower risk (229 vs. 1,435 RPN score) per single test than M-XM. A-XM permitted an annual reduction of 21 million RPN compared with M-XM (Table 4). A-XM was estimated to have a shorter TAT (19.1 vs. 23.3 min, *P* < 0.0001) and shorter hands-on time (1.1 vs. 5.3 min, *P* < 0.0001) per single test than M-XM. A-XM permitted an annual saving of 15.1 months of daytime workers’ labor (2,656 hrs of hands-on time) compared with M-XM (Table 4).

**Table 3:** Risk and TAT in each process for A-XM and M-XM.

Steps	Process	Potential defect	Potential intervention	Consequence	Risk by FMEA				TAT (min:sec)	
					S	O	D	RPN	Single test ^a^	Batch test ^b^
A-XM	Total							229	19:06	24:18
1	Prepare worklist on the middleware	Middleware malfunction	Re-booting	Delay	1	1	1	1	00:06	00:42
2	Prepare the tube for the test	Contamination	Repeat	Delay	1	1	1	1	00:04	00:29
3	Prepare segment of BC	Wrong sample or amounts, splashing, missed addition	Repeat	Delay, WR	10	2	6	120	00:21	02:30
4	Label the barcode of BC to the tube	Wrong information, mislabeling	Relabel	Delay, WR	10	1	5	50	00:04	00:29
5	Add segment RBC to the tube	Wrong sample or amounts, missed addition	Repeat	Delay, WR	9	1	6	54	00:08	00:57
6	Put tubes (prepared and sample) to the instrument	Spill, mechanical error, wrong barcode position	Repeat, relabel	Delay, WR	1	1	1	1	00:02	00:14
7	A-XM and interpretation	Instrument failure	Retest	Delay	1	1	1	1	18:14	18:14
8	Verify XM results on the middleware	Data transfer error (computational)	Re-transfer	Delay	1	1	1	1	00:06	00:43
M-XM	Total							1,435	23:15	42:42
1	Prepare the tube for the test	Contamination	Repeat	Delay	1	1	1	1	00:07	00:50
2	Manually write patient information on the tube	Wrong information	Relabel	Delay, WR	10	3	10	300	00:15	01:46
3	Prepare segment of BC	Wrong sample or amounts, splashing, forgot to add	Repeat	Delay, WR	10	2	6	120	00:17	02:02
4	Add 2 drops of patient serum	Wrong sample or amounts, wrong volume, fibrin or clot, forgot to add, RBC contamination	Repeat	Delay, WR	10	2	7	140	00:03	00:24
5	Make 2–5% RBC suspension with segment RBC	Wrong sample or amounts	Repeat	Delay, WR	9	2	6	108	00:15	01:43
6	Add RBC suspension to the tube	Wrong sample or amounts, forgot to add	Repeat	Delay, WR	9	1	6	54	00:02	00:15
7	Centrifuge for 15 sec	Spill, mechanical error	Repeat	Delay	5	2	1	10	00:38	04:24
8	Interpret saline phase result with manual recording	Incorrect reading, miswriting	Retest	Delay, FR	10	2	7	140	00:13	01:28
9	Add 2 drops of 22% bovine Alb	Wrong reagents or amounts	Repeat	Delay, WR	8	1	3	24	00:08	00:59
10	Water bath for 15 min at 37°C	Wrong work, uncontrolled temperature	Repeat	Delay, WR	7	2	5	70	15:10	46:09
11	Centrifuge for 15 sec	Spill, mechanical error	Repeat	Delay	5	2	1	10	00:40	04:40
12	Interpret Alb phase result with manual recording	Incorrect reading, miswrite	Retest	Delay, FR	10	2	7	140	00:12	01:25
13	Washing 3 times using the instrument for 3 min	Wrong work, instrument failure	Repeat	Delay, WR	7	2	3	42	03:16	22:54
14	Add 2 drops of AHG	Wrong reagents or amounts	Repeat	Delay, WR	8	1	1	8	00:10	01:09
15	Centrifuge for 15 sec	Spill, mechanical error	Repeat	Delay	5	2	1	10	01:03	07:21
16	Interpret AHG phase result with manual recording	Incorrect reading, miswrite	Retest	Delay, FR	10	2	7	140	00:13	01:34
17	Interpret final (3 phase integration) XM results	Incorrect interpretation	Repeat	WR	8	2	3	48	00:07	00:49
18	Manual input of results on LIS	Mistyping	Correction	WR	10	1	7	70	00:24	02:50

Abbreviations: BC, blood component; Alb, albumin; AHG, anti-human globulin; WR, wrong results; FR, false results; S, severity; O, occurrence; D, detection; RPN, risk priority number (S x O x D); FMEA, failure mode and effects analysis.

^a^ Numbers after the decimal place are rounded

^b^ In a batch, seven XM tests are performed.

**Table 4:** Comparison of quality and costs between A-XM and M-XM.

Description		A-XM	M-XM	*P* ^a^	Savings		
					per single test	per batch test	per year ^b^
**Quality improvement**	**RPN**	229	1,435		1,206	8,442	46 million
	**TAT**	19.1 min (19.0–19.2)	23.3 min (23.2–23.4)	< 0.001	4.2 min	29.4 min	2,656 hr
	**Hands-on time**	1.1 min (1.0–1.2)	5.3 min (5.2–5.4)	< 0.001	4.2 min	29.4 min	2,656 hr ^c^
**Cost-effectiveness**	**Cost** ^**d**^	1.44 USD	2.68 USD		1.24 USD	8.68 USD	47,042 USD

Abbreviations: See Table 1; RPN, risk priority number (S x O x D); TAT, turnaround time

TAT and hands-on time are shown as median (interquartile range).

^a^ *P* value is assessable only for TAT and hands-on time.

^b^ Annual savings are simulated based on 37,937 XM tests in 2018.

^c^ Equal to 15.1 months of working time, based on the average monthly working hours for daytime worker (176 hrs).

^d^ Cost = direct cost (consumables + reagents) + indirect cost (depreciation costs and labor costs).

Regarding cost, A-XM was estimated to have lower costs per single test than M-XM (1.44 vs. 2.68 USD) (Tables 4 and 5). A-XM had higher direct costs (1.02 vs. 0.67 USD) but lower indirect costs (0.42 vs. 2.03 USD) than M-XM. It was estimated that A-XM could save 47,042 USD annually compared with M-XM, with saved indirect cost (60,547 USD) and increased direct cost (13,505 USD). The calculated average operator salary per minute in the present study was 0.38 USD (429 Korean won) [36].

**Table 5:** Comparison of costs between A-XM and M-XM.

Cost category			A-XM		M-XM	
			Unit	Costs (USD)	Unit	Costs (USD)
**Direct cost / test**		** **		**1.02**		**0.67**
	Consumables	Serum separator	1	0.02	1	0.02
		Plastic tube	1	0.02	1	0.02
		Typesafe	1	0.28	1	0.28
		Poly Cassette	1/6	0.62	–	–
		Dilution trays	1/16	0.03	–	–
		Glass tube	–	–	1	0.04
		Dropper	–	–	1	0.02
	Reagents	BLISS	0.05 mL	0.05	–	–
		22% albumin	–	–	0.05 mL	0.05
		Anti-human globulin	–	–	0.10 mL	0.24
**Indirect cost / test**				**0.42**		**2.03**
	Labor cost ^a^		min of hands-on time	0.42	5.3 min of hands-on time	2.03
**Total cost = direct cost (consumables + reagents) + indirect cost (labor cost)**				**1.44**	** **	**2.70**

Abbreviations: A-XM, automated cross-matching; M-XM, manual cross-matching.

^a^ Labor costs were calculated on the basis of the average operator salaries and unit values [36]: the average operator salary per minute in this study was 0.38 USD (429 Korean won), and the annual rate of inflation was considered as 3%.

## Discussion

To the best of our knowledge, this study is the first to implement A-XM using VISION Max in a clinical transfusion laboratory. Laboratory automation is now expanding its reach into hematology, urinalysis [25–27], microbiology [39,40], special immunology [41], and even into blood banks [29,30]. While there is rapid implementation of automated blood grouping in clinical laboratories, automation of XM in clinical laboratories is in a very early stage [29,30]. The benefits of the A-XM using VISION Max, as seen in our results, were reduced risk, reduced TAT and hands-on time, and reduced cost through test automation.

**Fig 1:**
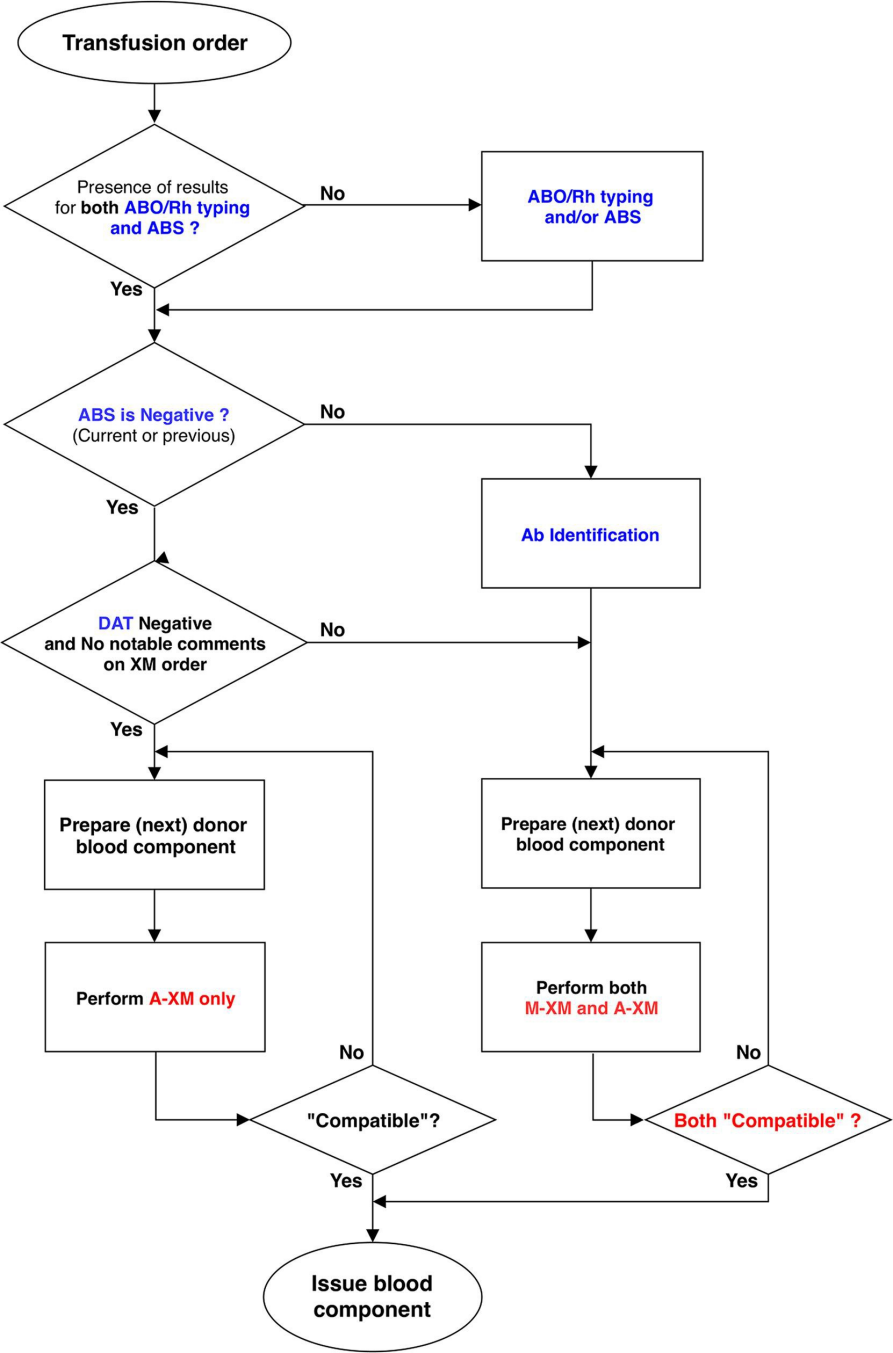
Suggested pre-transfusion workflow applied to the VISION Max middleware system. It was based on the comparative analyses and customized rules for the blood bank of Konkuk University Medical Center. Abbreviations: ABS, antibody screening test; DAT, direct antiglobulin test; A-XM, automated cross-matching; M-XM, manual cross-matching.

In addition to the direct benefit related to automation, the other biggest advantage of the VISION Max system is that its middleware system connects LIS and VISION Max bi-directionally; the middleware can review the previous results and other related orders of a patient and suggest a reflex test when necessary. In our study, all seven discordant cases were A-XM incompatible and M-XM compatible, suggesting that the A-XM can determine agglutination as positive more sensitively than visual inspection. The clinical significance of the additional positive A-XM results remains open. We expect the increase in safety will be achieved by adopting A-XM as shown in the risk assessment in this study. Based on the discordant results, the pre-transfusion workflow using VISION Max and middleware system was proposed (**Fig 1**). Since we start to adopt A-XM as first-line routine testing in clinical laboratories, we considered the introduction of A-XM conservatively and sequentially for patient safety. It was based on the comparative analyses and customized rules in the blood bank of KUMC. And in the presence of any factors that may affect RBC antigen-antibody immunologic response, we made a record of additional double-check by M-XM (as repeat test). Using the reflex test function of middleware, the A-XM is actively used as a routine laboratory practice. In the review of the data for 8 months after the introduction of A-XM workflow (**Fig 1**), there has been no discrepant result between A-XM and M-XM. We reviewed the additional specification of the incompatible result of A-XM after introduction into the routine. For eight months, a total of 7,235 A-XM were tested and among them, incompatible results were 5.00% (362/7,235). Among incompatible 362 results, ABS positivity was 57.2% (207/362). After accumulating more experience, we can adopt more efficient rules and reflex tests. However, even after implementing A-XM, M-XM is still being performed in a small number of samples as a comparative method when necessary or as an alternative in an emergency setting. If multiple units of packed RBCs are requested, performing both A-XM and M-XM simultaneously is more efficient than waiting for sequential results of A-XM; in an emergency, abbreviated M-XM can be performed, and then packed RBCs can be issued [1,3].

In this study, cost reduction was a noticeable, unexpected advantage. Before the introduction of VISION Max, there were some concerns regarding the cost increase. Actually, the individual cost of consumables is relatively higher in A-XM than in M-XM. Increase of direct costs, including consumables and reagents, was 0.35 USD/test costs (from 0.67 USD/test to 1.02 USD/test). However, the reduction in indirect costs was 1.59 USD/test due to the reduction of the medical technician’s hands-on time, which was much higher than the increase in direct costs (from 2.03 USD/test to 0.42 USD/test). The procedure for the additional positive results in A-XM should also be included for the assessment of the costs and the time required (repetition with M-XM, other packed red blood cells tested). But this was an irregular event and a very small proportion, we did not concern in comparison of cost and time. Contrary to the initial concerns, the total costs as well as hands-on time were reduced. By reducing the hands-on time spent on the XM testing, medical technicians could have more time to spend in improving the test quality or in performing other tests.

Another advantage of VISION Max system is data accumulation and easy statistics. If one XM testing is ordered, a medical technician performs the XM testing until he/she finds a compatible/least incompatible blood component. In M-XM, medical technicians run the following XM continuously leaving only a transient manual record, if the results are incompatible; therefore, it was impossible to identify the actual number of XM testing performed and to accumulate data in M-XM. However, for A-XM using VISION Max, the compatibility of each XM Poly Cassette is stored as an image with agglutination grade on a separate laboratory server from the hospital LIS, functioning as a backup. After the introduction of VISION Max at the blood bank of KUMC, the incompatible result of A-XM was 6.7% (199/2,954 tests) for two months from 1 October to 31 December, 2018.

This study has some limitations. In A-XM, only the 3rd phase (anti-human globulin phase) was performed, whereas in M-XM three phases were performed sequentially. Since 1984, the American Association of Blood Banks recommended that the full XM, including anti-human globulin phase, could be replaced by an abbreviated XM in patients with negative ABS [1,3]. According to each laboratory’s policy, abbreviated XM is performed worldwide [4,42–44]. If the result in each phase is inconsistent in full XM, results of the 3^rd^ phase determines compatibility in most cases [2]. We assumed that a different result in test phase may not have a big impact in practice. Additionally, in TAT analyses, time measurement was based on only seven XM tests, which is generally not a sufficient number for comparison; however, the TAT results were very similar with narrow ranges. Another limitation is that, considering the variation of laboratory and pre-transfusion testing across institutions, our estimation on quality and cost may not be extrapolated directly to other laboratories but can be used as one of the references. Further studies are needed for the blood bank automation including A-XM. In conclusion, this is the first attempt to implement A-XM using VISION Max in a clinical laboratory. Our multidimensional analysis showed that VISION Max A-XM is a safe, practical, and reliable alternative for pre-transfusion workflow with the potential to improve quality and cost-effectiveness in blood banks.

## Supporting information

S1 Table Description of the seven cases showing discordant results between M-XM and A-XM. (DOCX)

S1 File Standard operating procedure for XM in blood bank of Konkuk University Medical Center. (DOCX)
