# Supplementary material for: Benefits of VISION Max automated cross-matching in comparison with manual cross-matching: A multidimensional analysis
Source: PLoS One. 2019 Dec 23;14(12):e0226477. doi: 10.1371/journal.pone.0226477 (PMC6927601; doi:10.1371/journal.pone.0226477)
Supplement: S1 Table — (DOCX) [file pone.0226477.s001.docx]

**S1 Table. Description of the seven cases showing discordant results between M-XM and A-XM.**

| Case No | Demographic | | |  | Current unexpected Ab | |  | XM | |
| --- | --- | --- | --- | --- | --- | --- | --- | --- | --- |
|  | Age (yr) | Gender | ABO/Rh typing |  | Screening | Identification |  | Manual (grade ^a^) | Auto (grade) |
| 1 | 53 | Female | A+ |  | Negative | None |  | Compatible (0) | Incompatible (±) |
| 2 | 70 | Female | AB+ |  | Negative | None |  | Compatible (0) | Incompatible (±) |
| 3 | 52 | Male | AB+ |  | Negative | None |  | Compatible (0) | Incompatible (±) |
| 4 | 48 | M | A+ |  | Negative | None |  | Compatible (0) | Incompatible (±) |
| 5 | 48 | Female | AB+ |  | Negative | None |  | Compatible (0) | Incompatible (±) |
| 6 | 78 | M | AB+ |  | Positive | Anti-P_1_ antibody |  | Compatible (0) | Incompatible (±) |
| 7 | 1 | Female | AB+ |  | Positive | Anti-Le^a^ antibody |  | Compatible (0) | Incompatible (1+) |

Abbreviations: A-XM, automated cross-matching; M-XM, manuals-matching

^a^ Results of agglutination are determined as 0, ±, +1, +2, +3, and +4.
