# Supplementary material for: Benefits of VISION Max automated cross-matching in comparison with manual cross-matching: A multidimensional analysis
Source: PLoS One. 2019 Dec 23;14(12):e0226477. doi: 10.1371/journal.pone.0226477 (PMC6927601; doi:10.1371/journal.pone.0226477)
Supplement: S1 File — (DOCX) [file pone.0226477.s002.docx]

**STANDARD OPERATING PROCEDURE**

| **Department** | **Laboratory medicine, Blood transfusion services** |
| --- | --- |
| **Name of Procedure** | **Crossmatch, manual** |

1. **SCOPE AND APPLICATION**

This procedure is applied for compatibility testing of all patients requiring transfusion. Routinely major crossmatch is done in which donor red cells are crossmatched with patient serum/plasma to detect incomplete antibodies in the patient serum/plasma (including indirect antiglobulin phase). Incompatible blood units should not being used for transfusion.

1. **PRINCIPLE**

Red cells possess a variety of antigens, for identifying corresponding antibodies in the patient’s sample, donor red cells are tested against the patient’s serum/plasma or serum/plasma. The reaction between a specific antigen and its specific antibody is noticed by the presence of agglutination or hemolysis. Positive reaction in any test indicates incompatibility.

1. **SPECIMEN**

**3.1 PATIENT PREPARATION: NONE**

**3.2 SPECIMEN**

- Tube: Plaint tube without anticoagulant
- Type and amount: 5mL of venous whole blood or 2-3 mL of serum/plasma.
- Storage: 4^0^C (± 2^0^C)
- Serum/plasma should be separated by g for 10 mins from whole blood, immediately.

**3.3 SPECIMEN REJECTION CRITERIA**

- Patient sample older than 24 hours from specimen collection
- Patient sample without appropriate labeling (patient’s ID/name/age/gender and name of phlebotomist)
- Hemolyzed sample by visual inspection
- Not enough specimen less than 1.5 mL (as serum/plasma)

1. **MATERIAL and EQUIPMENT**

**4.1 MATERIAL**

- 0.9% Saline
- Polyspecific antihuman globulin reagent (anti-IgG+anti-C3d)
- 22% bovine albumin
- Anti-human globulin
- IgG sensitized control cells

**4.2 EQUIPMENT**

- Refrigerator to store samples & reagents at 4^0^C (± 2^0^C)
- Tabletop centrifuge
- Serofuge
- Cell washer

**4.3 GLASSWARE and OTHERS**

- Pasteur pipettes
- Tubes
- 12x75mm tubes
- Disposal box
- 2 glass beakers
- Dropper
- Aluminum racks to hold serum/plasma and tubes

1. **PROCEDURE**

**5.1 1st saline phase/room temperature immediate spin**

Saline room temperature is done to detect Major ABO incompatibility and complete (IgM) antibodies/cold antibodies like M, N, S, P, Lewis, Lutheran, etc. This crossmatching method can be done for the issuance of blood in emergencies.

1. Take a test tube.
2. Label with patient’s/donor ID on the tube.
3. Prepare 2-5% red cell suspension of donor red cells.
4. Dispense 2 drops of patient serum/plasma into the labeled tube.
5. Add one drop of donor red cell suspension (from step 3) to the tube containing patient serum/plasma.
6. Spin immediately at 1500g for 15 seconds.
7. Take out the tube gently.
8. Observe for hemolysis and then for agglutination by gentle shaking the tube.
9. Grade and record results, manually.
10. Always continue with anti-human globulin phase, even in emergencies. But in this case, blood units can be released after this phase.

**5.2 2nd albumin phase/37 ^O^C phase**

1. Add 2 drops of bovine albumin and incubate the tube for 30-45 minutes for albumin at 37^O^C
2. Take out tubes from 37^O^C.
3. Spin immediately at 1500g for 15 seconds.
4. Take out the tube gently.
5. Observe for hemolysis and then for agglutination by gentle shaking the tube.
6. Grade and record results, manually.

**5.3 3rd antiglobulin phase (AHG phase)**

1. Wash three times with 0.9% saline using cell washer for 3 mins.
2. Add 2 drops of anti-human globulin.
3. Spin immediately at 1500g for 15 seconds.
4. Take out the tube gently.
5. Observe for hemolysis and then for agglutination by gentle shaking the tube.
6. Grade and record results, manually.
7. **INTERPRETATION OF RESULT**
8. Compatible for transfusion: No Hemolysis / No Agglutination of red cells
9. Incompatible for transfusion: Hemolysis/Agglutination of red cells of any grade (trace, 1+,2+,3+,4+) / Mixed field.

NOTE:

- All steps should be done immediately
- Never use plastic tubes for crossmatch as it adsorbed IgG antibody which can lead to false negative results
- Shaking should be done gently
- Hemolyzed bag should not be selected for crossmatch
- Use clean glasswares
- Use all reagents according to the manufactures advice
- Never issue blood which is found incompatible at any phase of crossmatch

Limitations

- The saline/enzyme crossmatch will not:

Detect incomplete antibody

Ensure normal donor’s red blood cell survival

Detection of antibodies connected to low level presence of antigens (as with heterozygous expressed blood groups like Fy^a^/Fy^b^)

1. **DOCUMENTATION**

Enter results in laboratory information system.

All records are initialed by technician who performed the test and the technologist who has verified the result.

1. **RESPONSIBILITY**

It is the responsibility of the technician in the clinical laboratory to perform compatibility testing to demonstrate ABO compatibility and report the results. If any unexpected antibody is detected, the medical officer should be informed for further investigation.
